# Marker-Assisted Breeding of Improved Maternal Haploid Inducers in Maize for the Tropical/Subtropical Regions

**DOI:** 10.3389/fpls.2018.01527

**Published:** 2018-10-18

**Authors:** Vijay Chaikam, Sudha K. Nair, Leocadio Martinez, Luis Antonio Lopez, H. Friedrich Utz, Albrecht E. Melchinger, Prasanna M. Boddupalli

**Affiliations:** ^1^International Maize and Wheat Improvement Center (CIMMYT), Nairobi, Kenya; ^2^International Maize and Wheat Improvement Center (CIMMYT), Hyderabad, India; ^3^International Maize and Wheat Improvement Center (CIMMYT), Texcoco, Mexico; ^4^Institute of Plant Breeding, Seed Science and Population Genetics, University of Hohenheim, Stuttgart, Germany

**Keywords:** maize, doubled haploids, maternal haploid inducers, marker-assisted selection, phenotyping

## Abstract

For efficient production of doubled haploid (DH) lines in maize, maternal haploid inducer lines with high haploid induction rate (HIR) and good adaptation to the target environments is an important requirement. In this study, we present second-generation Tropically Adapted Inducer Lines (2GTAILs), developed using marker assisted selection (MAS) for *qhir1*, a QTL with a significant positive effect on HIR from the crosses between elite tropical maize inbreds and first generation Tropically Adapted Inducers Lines (TAILs). Evaluation of 2GTAILs for HIR and agronomic performance in the tropical and subtropical environments indicated superior performance of 2GTAILs over the TAILs for both HIR and agronomic performance, including plant vigor, delayed flowering, grain yield, and resistance to ear rots. One of the new inducers 2GTAIL006 showed an average HIR of 13.1% which is 48.9% higher than the average HIR of the TAILs. Several other 2GTAILs also showed higher HIR compared to the TAILs. While employing MAS for *qhir1* QTL, we observed significant influence of the non-inducer parent on the positive effect of *qhir1* QTL on HIR. The non-inducer parents that resulted in highest mean HIR in the early generation qhir1+ families also gave rise to highest numbers of candidate inducers, some of which showed transgressive segregation for HIR. The mean HIR of early generation qhir1+ families involving different non-inducer parents can potentially indicate recipient non-inducer parents that can result in progenies with high HIR. Our study also indicated that the HIR associated traits (endosperm abortion rate, embryo abortion rate, and proportion of haploid plants among the inducer plants) can be used to differentiate inducers vs. non-inducers but are not suitable for differentiating inducers with varying levels of haploid induction rates. We propose here an efficient methodology for developing haploid inducer lines combining MAS for *qhir1* with HIR associated traits.

## Introduction

Through *in vivo* induction of haploids and subsequent chromosomal doubling, genetically homozygous breeding lines can be produced in maize in just two crop seasons compared to the traditional inbreeding approach that requires 6–9 crop seasons. Fully homozygous lines obtained through the chromosomal doubling of haploids are referred as DH lines. DH lines when used in maize breeding programs help to increase the selection efficiency because of increased additive genetic variance, no dominance variation, no within-family segregation and increased precision to estimate genotypic value (Forster and Thomas, 2005; Seitz, 2005). Use of DH technology drastically reduces the “time to market” (Geiger and Gordillo, 2009), thereby allowing rapid deployment of improved maize hybrids.

Haploids can be produced in many plant species using *in vitro* methods like anther culture, microspore culture, gynogenesis and embryo rescue after wide hybridization (Forster and Thomas, 2005; Forster et al., 2007). Despite great efforts to establish anther culture in maize, it was not successfully adapted to produce DH lines as the response is highly genotype dependent and most genotypes are recalcitrant (Melchinger et al., 2005; Chang and Coe, 2009). However, unlike other crop species, maternal haploids can be produced in maize reliably and at reasonably high frequency through *in vivo* induction using specialized genetic stocks called haploid inducers. Coe (Coe, 1959) identified an inbred, Stock 6, which produced maternal haploids at relatively high frequency (1–3%). Identification of Stock 6 laid the foundation for an array of haploid inducers in maize. Haploid inducers with high HIR were developed based on Stock 6 in India (Sarkar et al., 1972, 1994; Aman and Sarkar, 1978), Russia (Zavalishina and Tyrnov, 1984; Shatskaya, 2010), China (Liu and Song, 2000), and France (Lashermes and Beckert, 1988). Based on Stock 6 derived inducers, more effective haploid inducers with high HIR (>6%) were developed in the last 15–20 years, which made commercial production of DH lines a possibility in maize. These inducers include RWS (Röber et al., 2005) and UH400^1^ in Germany, MHI in Moldavia (Chalyk, 1999), and the PHI series in Romania (Rotarenco et al., 2010). All these inducers possess *R1-nj* anthocyanin marker for haploid identification at seed stage. Recently inducers equipped with high oil trait were developed in Germany (Melchinger et al., 2013) and China (Dong et al., 2014), which aid automation of haploid identification (Melchinger et al., 2018).

Until recently, there were no tropical haploid inducers in maize with high haploid induction rates and acceptable agronomic performance. When the temperate inducers UH400 and RWS were grown in tropical conditions they showed similar haploid induction rates as in temperate environments but showed poor adaptation (Prigge et al., 2012a), including poor plant vigor, very early flowering resulting in non-synchrony with tropical germplasm, poor tassel characteristics, very low seed set, and extreme susceptibility to tropical diseases and ear rots. These result in difficulties in maintenance of these lines (Prigge et al., 2012a) and making them inefficient for use in large-scale haploid induction and derivation of DH lines in the tropics. To address these problems, CIMMYT in collaboration with the University of Hohenheim generated first-generation tropically adapted inducer line (TAIL) candidates (Prigge et al., 2012a), among which two best lines with high HIR along with their inducer hybrid were made available for maize breeding programs worldwide in 2012^2^. While these TAILs show relatively better agronomic performance compared to the temperate inducers (Prigge et al., 2012a), still there was significant scope to further improve these in terms of HIR, plant vigor, delayed flowering and susceptibility to tropical foliar diseases and ear rots. Therefore, the main objective of this study was to develop second-generation tropicalized haploid inducers (2GTAILs) that show high HIR and better agronomic performance than the TAILs, for wider adaptation and use in the maize DH breeding programs in the (sub)tropical regions.

One of the limitations in improved haploid inducer development is that the trait “haploid induction” cannot be directly assessed using any specific morphological markers/traits present in the inducers; therefore, accurate determination of HIR requires crossing of the putative inducer candidates with testers having recessive phenotypes, such as *liguleless* and *glossy* (Lashermes and Beckert, 1988; Röber et al., 2005; Prigge et al., 2012a) or by using herbicide resistance trait (Melchinger et al., 2016). However, conducting such testcrosses and evaluating the recessive phenotypes in the greenhouse is time-consuming and resource-intensive (Prigge et al., 2012b), limiting the number of families that can be evaluated for HIR. All the genetic studies undertaken so far to identify genomic regions conditioning the HIR revealed a major QTL, *qhir1*, on chromosome1 along with few other minor QTLs (Deimling et al., 1997; Barret et al., 2008; Prigge et al., 2012b). Very recently, the gene underlying this QTL on chromosome 1 (*qhir1*) has been identified as a sperm specific phospholipase, and was cloned and characterized (Gilles et al., 2017; Kelliher et al., 2017; Liu et al., 2017). It was also shown that *qhir1* causes segregation distortion and is selected against in the segregating families (Barret et al., 2008; Prigge et al., 2012b; Xu et al., 2013; Dong et al., 2014), thereby reducing the number of families with the haploid induction trait in conventional phenotypic selection. Therefore, in haploid inducer breeding, it was recommended to use MAS to maintain the *qhir1* allele, to improve selection intensity and to save time and resources involved in phenotyping for identification of plants or families with potentially high HIR (Prigge et al., 2012b; Dong et al., 2014). However, MAS for *qhir1* was demonstrated only in populations produced from crosses of temperate inducers with temperate non-inducer inbreds (Dong et al., 2014). Thus, the second objective of our study was to determine the effectiveness of MAS for *qhir1* in tropical inducer development using several populations derived from TAILs and elite tropical inbreds developed at International Maize and Wheat Improvement Center (CIMMYT).

Considering the time and resources involved in assessment of HIR, it would also be efficient to use any morphological traits known to be associated with high HIR to select plants or families that can lead to identification of inducers with high HIR. Recently it was shown that ears of individual plants and families with high HIR showed increased rates of endosperm and embryo abortion compared to plants or families with low or no HIR (Prigge et al., 2012b; Xu et al., 2013; Nair et al., 2017a). It is also well known that haploid plants do occur in the progenies of inducers. In fact, inducers like Stock 6 were identified based on the observation of high frequency of haploids in selfed progenies (Coe, 1959). The third objective of this study is to explore the possibility of using endosperm/embryo abortion rates and proportion of haploids in selecting inducer families with high HIR.

## Materials and Methods

### Germplasm Used

Three first-generation tropical haploid inducer lines (TAIL7, TAIL8, and TAIL9) and a temperate hybrid inducer (UH400 × RWS), all with the *R1-nj* marker (with distinct purple/red coloration in the aleurone of the endosperm as well as in embryo) were used as sources of haploid induction. These inducers also carry the purple stem marker. Six elite tropical inbred lines (developed by CIMMYT) with no apparent haploid induction ability were used as non-inducer parents. These inbreds differ in their adaptation, grain color and heterotic grouping, as indicated in **Table 1**.

**Table 1:** List of non-inducer parents used in the development of 2GTAILs.

Inbreds	Adaptation	Seed color	Heterotic group
CML269	Lowland tropical	White	B
CML451	Lowland tropical	Yellow	B
CML495	Lowland tropical	White	A
CML395	Subtropical	White	B
CKL05017	Subtropical	White	A
CK 05022	Subtropical	White	A

### Breeding Methodology

All the plant materials described below were planted in rows that are 4.5 m long and spaced at 75 cm. Each row accommodated 19 plants with a plant to plant spacing of 20 cm. Advancement of breeding crosses was done at CIMMYT’s Agua Fría experimental station in Mexico (20.26°N, 97.38°W; ∼110 masl) unless stated otherwise. Agua Fría is a lowland tropical location. Evaluation of candidate inducers was carried out in two other subtropical/mid-altitude locations, namely Metztitlán in Mexico (20.59°N, 98.76°W; ∼1328 masl), and Kiboko in Kenya (2.21°S, 37.72°E, ∼975 masl). Standard agronomic management recommended by CIMMYT was followed.

The four haploid inducers described above were used as pollen parents and were crossed to six different elite (sub)tropical non-inducer inbreds in the winter season of 2011 to generate 13 different F1 populations (**Supplementary Table 1**). Two breeding strategies were followed using the F1 families: the first strategy involved recurrent selfing starting from the F1 generation (F2 strategy), and the second strategy involved backcrossing the F1 populations to the respective non-inducer parent, followed by recurrent selfing (BC1 strategy). As detailed below, in both strategies, the focus in the early generations was on selection for *R1-nj* marker expression, agronomic performance, and MAS for *qhir1*, whereas in later generations, the emphasis was on selection for HIR. No selection was carried out for the purple stem color marker at any stage as it was shown to be of little use in haploid identification (Chaikam et al., 2016).

#### F2 Strategy

Each F1 family was planted in 10 rows in 2011 summer season, and advanced to F2 by selfing. Each F2 family was planted in 25 rows in the 2012 winter season and mass selection was practiced among F2 plants for agronomic traits, mainly for root and stem lodging, plant vigor, tassel size, resistance to foliar diseases, and synchrony in flowering with tropical germplasm. Ears from the selected F2 plants were scanned for *R1-nj* marker expression, eliminating ears with complete inhibition of the marker and/or diseased with ear rots. In total, 1458 F3 ears were selected, planted in ear-to-row in 2012 summer season and selected for agronomic traits and disease resistance among the families. Similarly, selection was practiced within the families, eliminating the plants with poor agronomic traits. In total, 440 agronomically best performing families with good expression of *R1-nj* marker were selected and advanced to F4 generation.

The bulked seeds for each of the F4 families were planted in a single row in the 2013 winter season, and leaf tissue was collected from all the plants in each family. DNA was extracted from bulked tissue as described earlier (CIMMYT, 2005). Genotyping for *qhir1* was performed with the flanking SSR markers *umc1917* and *bnlg1811* (Barret et al., 2008; Prigge et al., 2012b). Of the 440 families genotyped, 72 families carried the *qhir1* allele from inducer parent in homozygous condition and are designated as qhir1+. Majority of the families genotyped carried *qhir1* allele from non-inducer parent either in homozygous condition (qhir1-) or in heterozygous condition (qhir1+/-). 68 qhir1+ and 95 qhir1- F4 families with good agronomic traits were advanced to F5 generation. All these F5 families were planted in single rows in the 2013 summer season and crossed to a *liguleless* tester. Sufficient testcross seeds (>200) was obtained in 137 families for evaluation of HIR and to ascertain the effect of *qhir1*. In total, 17 families with >5% HIR (all being qhir1+) were advanced to the F6 generation in the 2014 winter season. Multiple ears were chosen from each family and were planted ear-to-row. In total, 75 F6 lines were evaluated in the 2014 summer season based on HIR and 9 families that showed high HIR were advanced to F7 generation and selected as candidate inducer lines. These lines were tested for HIR and agronomic performance in five different environments. These included the 2014 summer at Metztitlán (MZ14B), the 2015 winter at Metztitlán (MZ15A), 2016 winter in Agua Fría (AF16A) and Metztitlán (MZ16A), and 2016 Season A in Kiboko, Kenya (KI16A). Three lines that consistently showed high HIR (>8%) were selected as 2GTAILs and were further evaluated in 2017 winter at Agua Fría (AF17A).

#### Backcross1 (BC1) Strategy

F1 crosses described in **Supplementary Table 1** were backcrossed to the respective non-inducer parent (except the CML395/TAIL8 cross) to develop 12 BC1F1 families in the 2011 summer. Each BC1F1 family was planted in 10 rows in 2012 summer, plants were selected for agronomic traits as described in the F2 selfing strategy and selected plants were selfed. Each selfed ear from the selected plants was checked for *R1-nj* marker expression, selecting ears with partial or complete expression of Navajo phenotype to generate a total of 318 BC1F2 families. Bulked seeds from each of the BC1F2 families was planted ear-to-row in the 2013 winter. Leaf tissue was collected from each plant in a family, and the tissue was bulked. Genotyping for *qhir1* region was done by using two SSR primers flanking *qhir1*, namely *bnlg1811* and *umc1917* and three internal markers AY110477, X93 and X291 (Barret et al., 2008; Dong et al., 2013). Only six families were found to be homozygous qhir1+ for all the markers tested, and 174 families were homozygous qhir1–. The rest of the families were heterozygous or recombinants within the *qhir1* region. Multiple ears were selected in families that are homozygous and heterozygous for qhir1+. In total, 250 ears representing BC1F3 lines were selected and planted ear-to-row in the 2013 summer. Leaf tissue was collected from individual plants and the DNA was assayed with four markers (*bnlg1811, umc1917*, X93, and X291) in the *qhir1* region. Among the 4050 plants genotyped, 210 plants were homozygous qhir1+. Ears were harvested from 176 qhir1+ plants and 96 qhir1- plants and a total of 272 families were advanced to the BC1F4 generation. Each BC1F4 family was planted ear-to-row in the 2014 winter and was crossed to the *liguleless* tester. Of these, 47 lines showed high HIR; multiple ears were selected from each of these families and advanced to BC1F5. A total of 168 BC1F5 lines were evaluated for HIR in the 2014 summer at Metztitlán of which 27 lines with >7% HIR were selected and advanced to BC1F6. These 27 families were selected as candidate inducers and were evaluated in MZ15A, MZ16A, AF16A, KI16A, and AF17A. Of these, five best lines that consistently showed high HIR and good agronomic performance were selected as 2GTAILs.

#### Assessment of HIR Based on *lg* Tester

HIR was assessed by testcrossing the early generation families and advanced inbred lines from both F2 and BC1 strategies to *lg* tester hybrid PDH3 × PDH9 (Prigge et al., 2012a; Chaikam et al., 2016). The tester was stagger-planted four times at 5 days interval to achieve synchrony with the families and lines tested for HIR. In early generation F4 and BC1F4 families, bulked pollen from a single row of the family was used to cross 10–15 plants of the *liguleless* tester. During the MZ14B, MZ15B, MZ17B, KI16A and MZ17A evaluations, 9 candidate inducer lines from the F1 strategy and 27 candidate inducer lines from the BC1 strategy were grown in a single replication in two rows each. In the AF16A and MZ16A, a total of 46 entries comprising all 36 candidate inducers, 2GTAIL hybrid 2GTAIL009 × 2GTAIL006, selected TAILs, TAIL hybrids, temperate inducers and temperate inducer hybrid were planted in three replications in a α- lattice design. In AF16A, 10 CIMMYT Maize Lines (CMLs) that represent non-inducers were also planted in a separate trial in a randomized complete block design for HIR assessment. Bulked pollen of plants from each entry in each replication was used for crossing to the *lg* tester. Testcross ears obtained from the same inducer/non-inducer genotypes in a replication were bulked, shelled and good quality testcross seeds were selected by eliminating the seeds that were abnormal (endosperm aborted or embryo aborted or very small kernels) and infected by weevils and ear rots.

In early generation families of F4 and BC1F4, 200 testcross seeds were chosen as cutoff as recommended earlier (Prigge et al., 2012a). However, in the majority (67.2%) of the F4 and BC1F4 families more than 500 testcross seeds were obtained and used for HIR assessment (data not shown). In advanced generations with candidate 2GTAILs, TAILs, temperate inducers and non-inducers, 800–1000 testcross seeds were used for HIR evaluation. Testcross seeds were planted in styroform trays with soil that accommodated 200 seeds each. After 10–12 days of germination, the total number of seedlings germinated and the number of seedlings with *lg* phenotype were counted in each tray. HIR was determined in each tray separately as total number of *liguleless* plants/total number of plants evaluated and multiplied with 100.

#### Assessment of HIR Based on Breeding Populations

To determine the HIR of the selected 2GTAILs in breeder-relevant germplasm, seven populations were sourced from different breeding programs in CIMMYT, Mexico, and were planted in a randomized complete block design in three replications for making the testcrosses in MZ16B. Among these, three populations were developed for highlands, two were subtropical and two were lowland tropical. Each population was planted in six rows. 2GTAIL inducers 2GTAIL006, 2GTAIL009, and 2GTAIL009 × 2GTAIL006 were stagger-planted at 5 days interval for four times to achieve flowering synchrony with the populations. Pollen was bulked from 10–15 inducer plants and was applied to one row of the population. Even though all the crosses showed expression of the Navajo marker, it was not used for HIR evaluation as it can lead to significant false positives and false negatives (Röber et al., 2005; Prigge et al., 2011; Melchinger et al., 2014; Chaikam et al., 2016). Instead, we used adult plant characteristics of haploids like reduced plant vigor, erect leaves, and male sterility to distinguish them from diploids; such characteristics have been used as a “gold standard” in several studies (Melchinger et al., 2014; Chaikam et al., 2016). The induced seeds from the same cross in a replication were bulked from different ears and 500 good quality seeds per replication were planted in observation plots. Ploidy status of each surviving plant was ascertained based on the adult plant traits described above. Mean HIR of an inducer in a specific population was calculated based on HIR data from three replications involving a specific population.

#### Agronomic Performance

Agronomic data collected from the trials conducted in MZ16A and AF16A were used for analysis. In these two environments, 46 inducers were planted in three replications in a α- lattice design as described earlier. Agronomic traits were scored for each entry in each replication. The traits plant height, ear height, plant aspect and days to anthesis (DTA) were measured on 10 plants per row, as described earlier (Chaikam et al., 2016). Tassel size was scored on a subjective scale of 1–5 based on all the tassels in a row, where score 1 represents very big tassel with many branches and a score 5 represents very small tassels with few branches. The number of tassel branches was counted on 10 plants in a row. For determining the normal kernel number per ear, 10 ears were selected in each row. Each ear was shelled separately and the seeds were separated into three categories namely (a) normal kernels with proper formation of endosperm and embryo, (b) endosperm aborted kernels, and (c) embryo aborted kernels. Number of normal kernels were counted in each ear; the count included even the seeds infected by ear rots or weevils with proper embryo and endosperm.

#### Scoring for Endosperm and Embryo Abortion

For studying the EnAR and EmAR, ears from 46 inducers and 10 non-inducers planted in AF16A for evaluation of HIR were used. Ten ears were randomly selected from each entry in each replication. EnAR and EmAR were determined in each ear as described earlier (Xu et al., 2013; Nair et al., 2017a). For comparison of degree of EnAR and EmAR among the inducers with different levels of HIR (determined based on the *lg* testcrosses in AF16A), inducers were categorized into three classes, namely (a) inducers with low HIR (<5%), (b) inducers with moderate HIR (5–8%), and (c) inducers with high HIR (with >8% HIR).

#### Proportion of Haploid Plants Among the Inducers and Non-Inducers

For studying the proportion of haploid plants among the inducer and non-inducer lines, a separate trial involving all 36 candidate inducers and 10 CMLs was planted in a α- latticedesign with three replications. For each entry in each replication, 95 seeds were planted in five rows. Plants were grown till flowering, and each plant was visually assessed for plant vigor, leaf erectness, and tassel fertility, to determine the ploidy status, as described earlier (Melchinger et al., 2014; Chaikam et al., 2016). Since the seeds of the same origin were used for the trial to determine the HIR and agronomic performance in the same evaluation season (AF16A), we used the HIR data obtained from that trial for testing the relationship between the HIR and proportion of haploid plants. For comparison of proportions of haploid plants among the inducers with different levels of HIR, inducers were categorized into three classes of low, moderate and high HIR, as described above.

#### Validation of Effectiveness of MAS and Endosperm Abortion in Selection for HIR

To determine the effectiveness of using MAS and endosperm abortion trait in selection for HIR in early generations, the F2 population developed from a cross between the tropical line CKDHL0159 and 2GTAIL006 was chosen. A set of 5000 F2 plants were grown in AF15A and of these, 3809 plants were selected after eliminating agronomically poor plants. Leaf tissue was collected from each of the selected plants, and was genotyped using the KASPAR assays for SNPs PZA00714_1, PZE-101081177, SYN25793, SYN26730 (Hu et al., 2016) at LGC Genomics, United Kingdom. From this analysis, a total of 796 plants were identified to be qhir1+. Ears from qhir1+ plants and qhir1- plants were separated, and each ear was visually assessed for endosperm abortion. Less than 2% of the ears from qhir1– families showed some degree of endosperm abortion. Fifteen F2 ears were selected from each of the following categories of plants: (a) qhir1+ plants with endosperm abortion (qhir1+/EnA+), (b) qhir1– plants with no apparent endosperm abortion (qhir1-/EnA-), and (c) qhir1– plants with endosperm abortion (qhir1-/EnA+). Each of these ears were scored for endosperm abortion on a scale of 1–5, where score 1 represents no abortion and score 5 represents complete abortion of all the kernels. Bulked seeds from each ear were planted in two rows along with the *lg* tester in AF16A for HIR assessment. HIR was assessed using the same procedure, as described above.

### Statistical Analysis

Data was analyzed in two stages. At the first stage, entry means were calculated for (a) HIR based on the data obtained from each tray, (b) for agronomic traits based on the data collected on individual plants/ears, and (c) for EnAR and EmAR based on the data collected from individual ears. In the second stage, standard errors were estimated for entry means or different inducer group means (2GTAILs, TAILs, non-inducers) by ANOVA over replications and environments. The rates (HIR, EnAR, EmAR) were analyzed separately for the inducer group and non-inducer groups due to heterogeneous variances. To handle the different numbers of replications, environments, or entries, ordinary *t*-tests with a significance level of *P* < 0.05 (Snedecor and Cochran, 1989) were used. All the analyses were done using PLABSTAT (Utz, 2001), except that an additional ANOVA was conducted for the HIR data to determine the relative importance of different sources of variation in a random model by ASREML (Gilmour et al., 2008).

## Results

### MAS for *qhir1*

**FIGURE 1:**
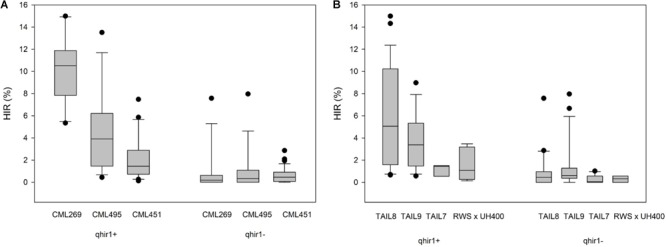
HIR in F5 families with qhir1+ and qhir1– genotypes involving **(A)** three different non-inducer parents; and **(B)** four different inducer parents.

The effect of *qhir1* on HIR was assessed in F5 families derived from the F2 strategy. In crosses involving the three lowland tropical inbreds (CML269, CML495, and CML451) the median HIR of the qhir1+ families was substantially higher compared to the qhir1- families (**Figure 1A**). qhir1- families involving these three parents showed very low mean HIR ranging from 0.6 to 1.2%. However, some qhir1– families showed HIR similar to qhir1+ families even though the frequency of such families is very low. In qhir1+ families, the mean HIR varied significantly among different non-inducer parents with a dramatic positive effect of *qhir1+* in crosses involving CML269 (**Table 2**). A significant positive effect of *qhir1* on HIR was also observed in crosses involving CML495 even though the mean HIR of qhir1+ families was about half of the mean HIR of qhir1+ families involving CML269. In CML451 crosses, *qhir1* showed only a marginal positive effect on HIR. While none of the qhir1+ families involving CML269 showed HIR < 5%, few families involving CML495 and CML451 showed HIR < 1% similar to that of most qhir1– families. In crosses involving the three subtropically adapted non-inducer parents (CML395, CKL05017, and CKL05022), only very few families were available at F5 stage for testing the effect of *qhir1* on HIR.

**Table 2:** Least square means of HIR in qhir1+ families of different non-inducer and inducer combinations.

	TAIL8	TAIL9	TAIL7	RWS × UH400	Mean†
CML269	10.8	5.3	–	–	7.6a
CML495	4.7	4.8	4.1	–	4.4b
CML451	2.4	2.6	1.2	1.6	1.9c
Mean^†^	6.0a	4.2a	4.1a	4.3a	4.7

***^†^**Means followed by the same letter are not significantly different at *P* ≤ 0.05.*

For all four inducers used in the crosses, qhir1+ families showed at least 2-fold higher median HIR compared to the qhir1– families (**Figure 1B**). qhir1– families showed very low mean HIR, irrespective of the inducer, ranging from 0.2 to 1.4%. Among the four inducers, qhir1+ families involving TAIL8 as an inducer parent showed highest mean HIR but differences among inducers were not significant (*P* < 0.05) (**Table 2**).

### Selection for Agronomic Traits and HIR

**FIGURE 2:**
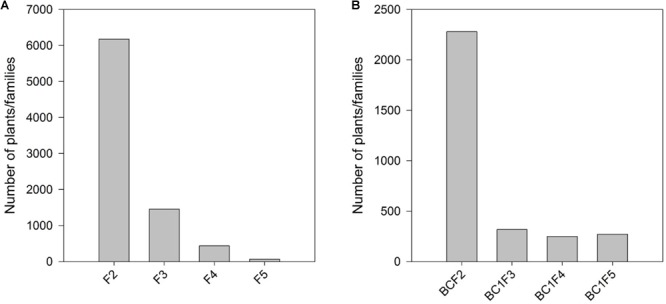
Number of plants/families grown and advanced in early generations after selection for agronomic traits and *R1-nj* marker expression in **(A)** the F2 strategy; and **(B)** the BC1 strategy.

The number of families selected at each stage involving each non-inducer parent starting from F2/BC1F1 generations till the selection of candidate inducers are presented in **Supplementary Table 2**. Phenotypic selections for agronomy and *R1-nj* marker expression resulted in elimination of 84.6% families by generation F4 in the F2 strategy (**Figure 2A**; **Supplementary Table 2A**), and of 71.5% families by generation BC1F2 in the BC1 strategy (**Figure 2B**; **Supplementary Table 2B**). Early generation families derived from crosses involving the three subtropical inbreds showed higher susceptibility to foliar diseases and ear rots (data not shown). Hence, selection for superior agronomic performance in F2, F3 families and BC1F1 families resulted in elimination of highest number of families in crosses involving the subtropical inbreds. This can be inferred from the observation that only 2.3% of the F2 plants involving the subtropical inbreds resulted in F4 families, while 9.3% of the F2 plants involving lowland tropical inbred lines resulting in F4 families. In the BC1 strategy too, crosses involving the three lowland tropical inbreds resulted in a higher proportion of BC1F2 families (15.7%) compared to the subtropical inbreds (8.6%) after selection for agronomy and *R1-nj* expression. All four inducers used in the crosses gave rise to similar proportion of lines after selection for agronomy in both the F2 and BC1 strategies (**Supplementary Table 3**). Subsequently to the selection for agronomic traits and *R1-nj*, MAS for *qhir1* aided in elimination of all qhir1– families in F4, BC1F2 and BC1F3 generations thereby reducing the number of families needed to be evaluated for HIR.

**FIGURE 3:**
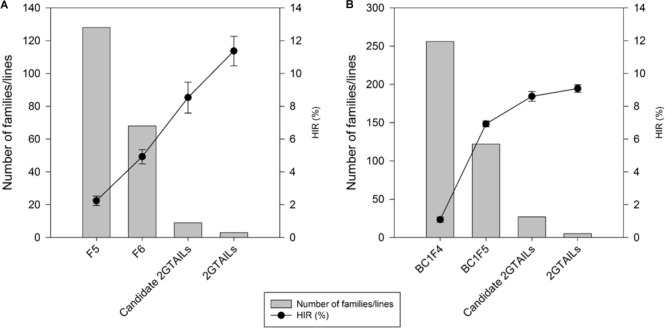
Number of families/lines advanced and evaluated for HIR and the improvement of HIR with selection in the advanced generations of **(A)** the F2 strategy; and **(B)** the BC1 strategy.

A steady increase in the HIR can be noticed with the advancement of generations in both F2 and BC1 strategies when comparing the HIR of families of different generations assessed in different evaluation cycles (**Figure 3**). F5 and BC1F4 families showed very low average HIR of 2.3% and 1.3%, respectively. In F5 and BC1F4 generations, the families assessed for HIR included both qhir1+ and qhir1– genotypes. In F6 and BC1F5 generations, which included only qhir1+ families, the HIR was more than double compared to earlier generation. In F7 and BC1F5 generations, elimination of families with low HIR and selection of families with high HIR as candidate inducers increased the HIR by another 2-fold compared to earlier generations. Further evaluation of candidate inducers in multiple locations and selection of lines that consistently showed high HIR and superior agronomic performance led to a further increase in the HIR by 33.2% and 5.6% in the final selected inducer lines from F2 and BC1 strategies, respectively.

### Composition of the Selected Candidates, 2GTAILs and Their HIR

The selected candidate inducers, the cross from which they were derived, and the HIR assessed based on *lg* tester in six environments was presented in **Supplementary Table 4**. The candidate inducers included nine lines derived from the F2 strategy, and 27 lines from the BC1 strategy. Among the non-inducer parents, CML269 contributed to a maximum number of candidates (25) followed by CML494 (7). CML395 and CKL05017 contributed to two candidates each. No candidate inducers were derived from crosses involving CML451 and CKL05022. Among the inducer parents, TAIL9 contributed to 20 inducers, TAIL8 to 16, and TAIL7 to 1. Temperate hybrid inducer, RWS × UH400, did not contribute to any candidate inducers. The majority of the candidate inducers (29 of 36) showed mean HIR similar to or higher than the inducer parents used. ANOVA for HIR data across environments indicated that inducers and the residual error accounted for most of the variation in HIR, as compared to inducer x environment interactions (**Table 3**).

**Table 3:** Estimates of variance components (Var) and their standard errors (SE) for HIR of thirty six candidate inducers evaluated across six environments.

Random effect	Var	SE
Environment	1.4	1.0
Inducer	2.2	0.6
Environment × Inducer	0.6	0.2
Residual error	1.9	0.2

From the 36 candidate 2GTAILs, eight inducers with high HIR and superior agronomic performance (as compared to TAILs) were selected as final 2GTAILs (**Table 4**). Among the selected 2GTAILs, six were derived from crosses involving CML269 and two were derived from crosses involving CKL05017. Three of the finally selected 2GTAILs were derived from the F2 strategy, and the rest from the BC1 strategy. The HIR of the 2GTAILs was presented in **Table 4** in comparison to the HIR of TAILs and temperate inducers. 2GTAILs showed significantly higher HIR compared to TAILs, while the HIR of 2GTAILs was not significantly different from temperate inducers. The 2GTAILs, on average, showed 47.8% increase in HIR compared to TAILs and a nominal 5.3% increase in HIR compared to the temperate inducers. Among all the inducers assessed for HIR including 2GTAILs, TAILs and temperate inducers, 2GTAIL006 showed the highest mean HIR (13.1%). 2GTAIL007 and 2GTAIL009 also showed high mean HIR of 10.1 and 10.9% respectively, which is comparable to the mean HIR of temperate inducer RWS. Other five selected 2GTAILs showed mean HIR ranging from 8.2 to 9.5%, which is significantly higher than the mean HIR of TAILs.

**Table 4:** HIR and agronomic performance of 2GTAILs in comparison to TAILs and temperate inducers.

Inducer	HIR			Agronomic traits						
	N¶	Mean	*SE*	Plant vigor	Plant height	Days to anthesis	Tassel size	Tassel branches	Normal kernels per ear	Ear rot
				Score 1–5	cm	days	Score 1–5	Number	Number	Score 1–5
**2GTAILs**										
2GTAIL006	7846	13.1	0.47	2.1	166.0	71.2	3.1	8.1	107.0	2.3
2GTAIL007	8453	10.1	0.42	1.8	190.1	70.0	3.2	7.1	71.5	3.2
2GTAIL009	7692	10.9	0.68	2.5	137.5	69.5	2.3	6.3	116.7	2.5
2GTAIL102	7686	9.2	0.45	1.8	165.7	71.4	1.7	13.7	105.1	1.9
2GTAIL104	7074	9.1	0.77	2.0	144.8	74.2	1.1	15.9	87.0	1.6
2GTAIL105	9675	9.4	0.99	2.6	147.2	73.2	2.3	10.5	89.6	1.7
2GTAIL109	7156	9.5	1.02	2.0	164.5	77.8	1.6	11.4	93.1	2.2
2GTAIL114	8187	8.2	0.46	1.2	165.4	75.2	1.2	12.7	110.5	2.3
2GTAIL009 × 2GTAIL006	5467	9.4	0.63	1.0	181.3	64.6	1.3	9.4	191.0	1.5
**Mean**^†^	7693	9.9a	0.23	1.9a	162.5a	71.9a	2.0a	10.6a	107.9a	2.1a
**TAILs**									
TAIL7	5116	7.1	0.63	3.1	128.8	64.4	2.8	11.2	63.8	3.8
TAIL8	5103	6.8	0.74	3.2	133.6	64.0	2.8	10.7	73.1	2.9
TAIL9	4589	6.6	0.72	3.4	120.2	62.2	3.3	5.5	95.7	2.9
TAIL9 × TAIL8	5240	6.4	0.16	2.8	141.3	61.9	2.5	8.8	130.9	2.6
**Mean**^†^	5012	6.7b	0.30	3.1b	131.0b	63.1b	2.9b	9.1b	90.9b	3.1b
**Temperate inducers**									
RWS	3405	10.6	1.01	3.8	88.1	59.2	3.6	10.7	57.0	2.8
UH400	4981	7.1	0.16	3.5	109.1	59.7	2.6	11.3	37.6	2.5
RWS × UH400	4801	10.6	1.42	3.2	121.6	53.0	2.6	16.5	112.8	2.0
**Mean**^†^	4396	9.4a	0.58	3.5b	106.3c	57.3c	2.9b	12.8c	69.1c	2.4ab

*^†^Means followed by the same letter are not significantly different at *P* ≤ 0.05. ^¶^Number of testcross seedlings evaluated in determining the HIR across all the environments.*

To validate if the HIR determined by using the *lg* tester can be representative of the HIR in germplasm relevant for the maize breeding programs, HIR of the two best 2GTAILs and their hybrid inducer was determined in three highland, two subtopical and two lowland tropical populations based on differences in adult plant traits between haploids and diploids (**Supplementary Table 5**). Similar to HIR tests using the *lg* tester, 2GTAIL006 showed the highest mean HIR of 13.6% across all populations. The mean HIR of 2GTAIL009 was 1.2% lower compared to the *lg* tester. The hybrid between 2GTAIL006 and 2GTAIL009 also showed a HIR consistent with *lg* tester.

### Agronomic Performance of the Selected 2GTAILs

The agronomic performance of 2GTAILs was significantly superior (*P* < 0.05) to the TAILs and temperate haploid inducers (**Table 4**). The 2GTAILs showed superior plant vigor (average score of 1.9), compared to the TAILs (average score of 3.1) and temperate inducers (average score of 3.5). Individually, each of the 2GTAILs showed better plant vigor compared to TAILs and temperate inducers. Also, the 2GTAILs on average showed 24 and 52.9% increase in plant height compared to TAILs and temperate inducers, respectively. Individually, all the 2GTAILs showed > 25% increase in plant height compared to TAILs, except 2GTAIL009 which had similar plant height as the TAILs. 2GTAILs also showed increased ear height (to the extent of > 43.3%) compared to the TAILs (data not shown). On average, 2GTAILs showed delayed anthesis by 8.8 days as compared to TAILs, and 14.6 days as compared to temperate inducers. Individually each of the 2GTAILs showed delayed flowering by at least one week compared to TAILs, whereas 2GTAIL109 and 2GTAIL114 showed up to 2 weeks delayed anthesis. Most of the 2GTAILs also possess considerably bigger tassels than the TAILs and temperate inducers. The average number of tassel branches was higher in temperate inducers followed by 2GTAILs. However, the tassel branches were much smaller in temperate inducers as indicated by the tassel size score. Among the 2GTAILs, 2GTAIL006, 2GTAIL007, and 2GTAIL009 have fewer number of tassel branches. Grain fill, as indicated by the normal kernels per ear, was also higher in the 2GTAILs to the extent of 18.7 and 56.2% compared to TAILs and temperate inducers, respectively. Individually, all 2GTAILs except 2GTAIL007 and 2GTAIL104 showed higher normal kernels per ear compared to the TAILs and temperate inducers. The 2GTAILs, except 2GTAIL007, showed higher resistance to ear rots compared to TAILs and temperate inducers. Among all the 2GTAIL inbreds, 2GTAIL114, and 2GTAIL102 showed the best agronomic performance. The inducer hybrid 2GTAIL009 × 2GTAIL006 showed excellent plant vigor, bigger tassel with more tassel branches compared to the parents and similar flowering date as the TAILs.

### Exploring the Use of HIR Associated Traits in Improved Inducer Development

The 46 inducers comprising the entire 36 candidate 2GTAILs, three TAILs, two temperate inducers and five hybrid inducers showed an average HIR of 6.4% while the 10 non-inducers showed an average HIR of 0.1% in *lg* testcrosses (**Table 5A**). The inducers showed very high EnAR of 30.7% compared to the 0.9% in non-inducers (**Table 5A**). Inducers also showed substantially higher EmAR of 5.3% compared to no EmAR in non-inducers (**Table 5A**). Among the 36 Inducers and 10 non-inducers, inducer plants showed a significantly higher proportion of haploid plants (7.9%) in the field compared to non-inducers (0.8%) (**Table 5B**). In addition, we explored if the HIR associated traits, namely EnAR and EmAR can potentially distinguish haploid inducers with varying levels of HIR. For this, we categorized the inducers with different levels of HIR and tested their association with the HIR-associated traits. Within the inducers, inducers with low, moderate and high levels of HIR did not show significant differences for the proportion of haploid plants. For EnAR, inducers with moderate and high levels of HIR did not differ significantly (*P* < 0.05), but both categories showed higher values than the inducers with low levels of HIR. For EmAR, all three inducer categories differed significantly from each other.

**Table 5:** HIR associated traits of **(A)** EnAR and EmAR, and **(B)** proportion of haploid plants in inducers and non-inducers and in inducers with different levels of HIR.

(A)				
		Mean^†^		

Lines	N	HIR	EnAR (%) ± SE	EmAR (%) ± SE
Inducers < 5% HIR	11	3.8	27.4a ± 1	4.4a ± 0.2
Inducers 5–8% HIR	25	6.4	31.8b ± 0.7	5.4b ± 0.2
Inducers > 8% HIR	10	9.2	32.9b ± 0.9	6.2c ± 0.2
Total	46	6.4	30.7 ± 0.5	5.3 ± 0.1
CMLs (non-inducers)	10	0.1	0.9 ± 0.2	0 ± 0
**(B)**				
	**Mean^†^**		

	**N**	**HIR**	**Haploid plants (%) ± SE**

Inducers < 5% HIR	11	3.8	7.2a ± 0.2
Inducers 5–8% HIR	18	7	8.2a ± 0.2
Inducers > 8% HIR	7	9.2	7.1a ± 0.3
Total	36	6.2	7.9 ± 0.1
CMLs (non-inducers)	10	0.1	0.8 ± 0.2

***^†^**Means followed by the same letter are not significantly different at *P* ≤ 0.05.*

### Validation of Using MAS and Endosperm Abortion in Selection for HIR

**FIGURE 4:**
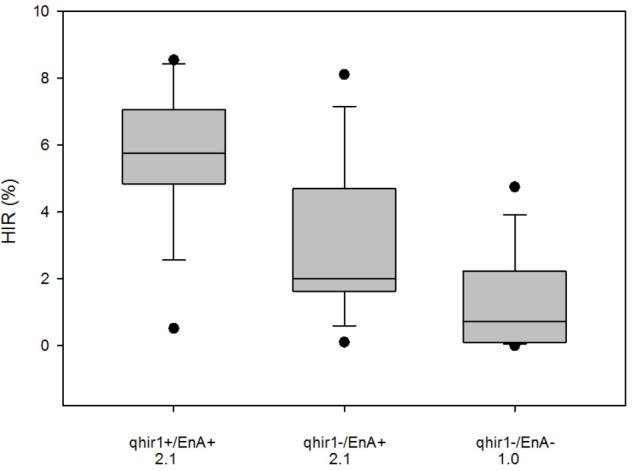
HIR of three F3 categories of plants selected from the F2 population CKL0159 x 2GTAIL006. Fifteen plants were assessed for HIR from each category. qhir1+/qhir1– indicates presence/absence of QTL *qhir1* in the F2 plants. EnA+/EnA– indicates presence/absence of endosperm abortion in the F2 ears. Mean values for endosperm abortion score were indicated for each category on the X-axis.

Genotyping 3809 F2 plants derived from the cross involving 2GTAIL, 2GTAIL006 and non-inducer line CKDHL0159 led to identification of 796 plants that were homozygous for qhir1+, 1135 plants homozygous for qhir1–, while the rest were either heterozygous or recombinants within the *qhir1* region. Significant segregation distortion was observed between the qhir1+ and qhir1– genotypes (Chi-square value = 59.51). Visual assessment of qhir1+ and qhir1– F2 ears indicated that ears from plants with genotype qhir1+ always showed some level of endosperm abortion, while the majority of qhir1– plants produced ears with no endosperm abortion. Very few qhir1– ears (<2%) showed some level of endosperm abortion similar to the families that are qhir1+. Based on qhir1 genotype and the endosperm abortion, plants were categorized into three classes. Mean scores for endosperm abortion were the same for qhir1+/EnA+ and qhir1–/EnA+ classes, which was more than double compared to qhir1–/EnA– class. Phenotypic distribution of HIR in 15 plants from each of the three phenotypic classes (**Figure 4**) revealed that the qhir1+ /EnA+ showed substantially higher median HIR compared to other two classes. Most qhir1+/EnA+ plants (11 out of 15) showed high HIR (>5%) while only three plants from qhir1–/EnA+ and one plant from fqhir1-/EnA- showed HIR > 5%. qhir1–/EnA+ families with a mean score of 2.1 for endosperm abortion showed almost double HIR compared to the qhir1-/EnA- families with no kernel abortion.

## Discussion

The maize crop is rapidly expanding in the tropical environments in terms of area cultivated and harvested (Alexandratos and Bruinsma, 2012; Phalan et al., 2013). DH technology could play an important role in rapid development and deployment of improved maize hybrids in the tropical environments. However, technical limitations like lack of adapted inducers with high HIR prevented large-scale adoption of the DH technology in the maize breeding programs (Prasanna et al., 2012; Prigge et al., 2012a). The 2GTAILs developed through this study have the potential to address an important need for haploid inducers with superior agronomic performance and high HIR especially in the (sub)tropical environments. To our knowledge, the 2GTAILs presented in this study combine the highest HIR and superior agronomic performance in (sub)tropical environments than ever reported. The best inducer line 2GTAIL006 consistently showed > 12% HIR, while the next best inducer 2GTAIL009 consistently showed > 9% HIR. The two lines combine well for making a inducer hybrid with far superior agronomic performance with a HIR that is intermediate to the HIR of the parental lines. Such high HIR in the second generation TAILs compared to the first-generation TAILs will have a significant positive effect on the efficiency of haploid induction, and consequently DH development. For example, the number of plants of a source population that need to be crossed to obtain a desired number of haploids can be reduced by 41.6% using the 2GTAIL with HIR of 12% compared to a TAIL with HIR of 7% (assuming that each ear produces at least 250 seeds). This reduction in plant count leads to considerable reduction in the field costs in terms of planting, agronomic management, pollinations and harvest. Further costs savings results in terms of processing the ears for haploid identification i.e., by dealing with significantly lesser number of seeds to identify desired number of haploids.

The two 2GTAILs with highest HIR (2GTAIL006 and 2GTAIL009) also exhibited superior agronomic performance in terms of plant vigor, delayed flowering for synchrony with tropical populations, normal kernels per ear, and resistance to ear rots, in comparison with the TAILs and temperate inducers. However, they have similar tassel characteristics as the TAILs. Several other 2GTAILs like 2GTAIL114, 2GTAIL102, 2GTAIL109 showed even far superior agronomic performance with very vigorous plants and tassels but showed lesser HIR compared to 2GTAIL006 and 2GTAIL009. If even superior agronomic performance is desired or if haploid inductions need to be conducted in harsh environments, we recommend using inducer hybrids such as 2GTAIL009 × 2GTAIL006 which showed outstanding performance in all aspects including tassel characteristics.

The superior agronomic performance of the 2GTAILs can further improve the efficiency in haploid inductions as such inducers may be used for large scale open pollinations in spatially or temporally isolated haploid induction nurseries, thus saving resources involved in manual pollinations. Currently, open pollination based haploid induction using first-generation TAILs is not feasible due to several reasons: (a) ear position of most tropical populations is higher than the tassel position of the TAILs; and (b) TAILs show poor pollen production. When using 2GTAILs as pollen parents in induction nurseries, it is also possible to achieve higher grain yields in the source populations compared to using TAILs for reasons cited above. Moreover, higher grain yield in the selfed ears of 2GTAILs, as compared to TAILs, helps in reducing the costs involved in multiplication of haploid inducer seed needed for induction nurseries.

Despite these improvements, there is an opportunity to further improve the accuracy and reliability of haploid identification when using 2GTAILs by integrating alternative marker systems like the red root marker (Chaikam et al., 2016) and high oil marker (Melchinger et al., 2013) as the *R1-nj* marker suffers from limitations like complete/partial inhibition, high portions of false positives and false negatives (Chaikam et al., 2014, 2016). Among the agronomic traits, tassel characteristics like size and the number of tassel branches can be further improved to achieve even higher pollen production. Further, 2GTAILs may be a good source to increase the HIR in tropical inducers. Some temperate haploid inducers with high HIR were identified as transgressive segregants in crosses between two inducer parents (Chalyk et al., 1994; Röber et al., 2005; Rotarenco et al., 2010). Thus, it may be possible to further improve the HIR in tropical inducers by identifying transgressive segregants with high HIR in crosses between different 2GTAILs.

Another important component of this study was to test different ways of developing haploid inducers efficiently. Firstly, we successfully demonstrated the utility of MAS for *qhir1* in development of improved haploid inducers. The *qhir1* locus has a significant positive effect on HIR and shows strong segregation distortion (Barret et al., 2008; Prigge et al., 2012b; Xu et al., 2013; Dong et al., 2014; Nair et al., 2017a). We started testing MAS early on in the haploid inducer breeding program, effectively making use of the *qhir1* linked SSR markers reported in earlier studies (Barret et al., 2008). Segregation distortion results in fewer genotypes with qhir1+ and far more genotypes that are qhir1– in the segregating populations. As shown here and other studies, most qhir1– genotypes have no/very low HIR (Prigge et al., 2012b; Dong et al., 2013, 2014; Nair et al., 2017a). Hence, use of MAS helps in effectively selecting qhir1+ genotypes that have higher HIR. In addition, several qhir1+ lines selected as 2GTAILs showed even higher HIR compared to the inducer parents used, indicating transgressive segregation. However, such transgressive segregants would be rare and most likely resulted from accumulation of other minor QTLs with positive influence on HIR (Prigge et al., 2012b). Hence, MAS may also favor identification of such transgressive segregants among qhir1+ genotypes. In our initial selections using SSR markers flanking the *qhir1*, we could observe a small proportion of parental lines which were not polymorphic for the flanking markers possibly due to disruption of linkage between the markers used in selection and the functional variant. This situation was further improved in later selections, when more tightly linked markers reported in other studies were used (Dong et al., 2013; Hu et al., 2016). The accuracy and efficiency of MAS for *qhir1* can be further improved now using the molecular markers designed to identify the causative 4 bp InDel polymorphism in the sperm specific phospholipase gene underlying the *qhir1* (Gilles et al., 2017; Kelliher et al., 2017; Liu et al., 2017). In addition to MAS for *qhir1*, breeding for improved inducers can also integrate MAS for *qhir8*, another QTL with significant positive effect on HIR after *qhir1* (Liu et al., 2015). This may further increase the selection intensity, reduce the number of genotypes needed to be phenotyped for HIR, and may lead to identification of inducers with potentially higher HIR.

Our study also revealed that the extent of positive effect of *qhir1* on HIR is influenced by both the non-inducer and inducer parents. Among the six non-inducer parents used, three subtropical non-inducer parents resulted in very few families to be tested for *qhir1* effect because of poor adaptation of early generation families resulting from these inbreds to lowland tropical conditions, where selections were carried out. Among the three-lowland tropical non inducer parents used, qhir1+ families involving the CML269 showed highest mean HIR followed by qhir1+ families involving the CML495. The qhir1+ families involving CML451 showed low mean HIR compared to CML269 and CML495. Aligned with these observations, CML269, followed by CML495, contributed to the largest number of candidate inducers. CML451 did not contribute to any candidate inducers even though it accounted for highest number of qhir1+ families phenotyped for HIR. In addition, several transgressive segregants with significantly higher HIR than the TAIL parents were identified only in crosses involving CML269, but not in crosses involving CML495. This could be because of a significantly higher positive effect of *qhir1* on HIR in families involving CML269, compared to CML495, as indicated by the mean HIR of qhir1+ families. Hence, the comparison of the mean HIR of qhir1+ families developed from different non-inducer parents could indicate, which non-inducer parent may lead to potentially larger number of inducers with high levels of HIR. Comparison of HIR of qhir1– families involving different non-inducer parents may not be a useful criterion as QTLs other than *qhir1* may contribute positively to increase the HIR in qhir1– families. Among the inducer parents used, even though the mean HIR is not significantly different among the four inducers, TAIL8 and TAIL9 contributed to more number of inducers than TAIL7 and RWS × UH400. This could be because of the poor agronomic performance in the advanced lines resulting from these inducers; TAIL7 is highly susceptible to ear rots and RWS × UH40 has poor adaptation to tropical environments. Hence, if multiple inducers (with no significant differences in HIR) are available, it would be better to choose inducers with better agronomic performance and adaptation to the target environment.

In addition to MAS for *qhir1*, the present study explored if phenotypic traits associated with the haploid induction trait can be used in developing new inducers. This study confirmed previous observations that endosperm abortion and embryo abortion occur in high frequency in the inducer ears compared to non-inducer ears (Xu et al., 2013; Nair et al., 2017b). Ears from qhir1+ plants always tend to show some level of abortion; while the majority of qhir1– families generally show no or very low levels of abortion (Nair et al., 2017a) and haploid plants occur at high frequencies among the inducer progenies (Coe, 1959) compared to no or very low levels of haploids in the non-inducers. Hence, while developing inducer lines, it would be pragmatic to select families/lines with these traits as they indicate presence of haploid induction capability. However, selection for endosperm abortion and proportion of haploid plants among the inducers may not necessarily result in identification of inducers with high HIR as these traits are not significantly different between inducers with different levels of HIR and, therefore, cannot distinguish inducers with varying levels of HIR. Embryo abortion showed significant differences among the inducers with different levels of HIR; hence, identification of plants/families with high level of EmAR may lead to identification of inducers with high HIR. However, estimating EmAR requires shelling of each ear, careful separation of embryo-aborted and non-aborted seeds, and recording their numbers. This is labor-intensive and may be difficult to practice on hundreds to thousands of ears resulting in early generations. Among all the three traits, only endosperm abortion can be scored quickly and efficiently to decide on the families/lines to be advanced with potential haploid induction capability before planting. As qhir1– families with endosperm abortion showed higher HIR compared to qhir1– families with no endosperm abortion, abortion is positively associated with HIR even in qhir1– families. This could be due to loci other than *qhir1* that positively influence HIR and cause abortion. Hence, we propose use of endosperm abortion as one of the useful criteria in addition to MAS for *qhir1* to eliminate most of the plants or families or lines that may have very low or zero HIR. If MAS for *qhir1* is not possible to apply for any reasons, selection of ears with some level of abortion at each advancement most likely leads to families or lines that have haploid induction capability. If the progenies from such ears show a higher proportion of haploids it is also a positive indication that the family/line has higher HIR than non-inducers. However, phenotypic tests for HIR still need to be carried out in the advanced generations to assess the HIR accurately and identify the lines with highest HIR. In addition, all the above mentioned traits are not preferred by maize breeding programs as abortion negatively affects grain yield, leading to difficulties in line maintenance and the haploid plants in inducer progenies result in waste of resources in induction nursery as the haploid plants are sterile and do not produce pollen. Considering these negative attributes, if high section pressure is applied against such traits, it may lead to families or lines with low or no HIR. Hence, these traits should be maintained at low to moderate levels.

Based on detailed observation and analysis of the breeding process followed in developing 2GTAILs, we suggest the following strategy for efficient development of improved haploid inducers:

(a) While selecting the inducer parents, if multiple options are available, choose inducers with better agronomic performance for the target location and decent HIR (≥6%). Since the non-inducer parent could have a pronounced effect on the HIR of the progenies derived by crossing with the inducer, we recommend selecting several non-inducer parents from opposite heterotic groups for making crosses with inducer parent(s).
(b) While selecting the non-inducer parents, emphasis should be given to agronomic traits, like plant vigor, bigger tassels, resistance to key diseases in the target agro-ecology and higher grain yield.
(c) As demonstrated in this study, haploid inducers with high HIR and good agronomic performance can be developed from both the F2 and BC1 strategies. However, if the inducer parent shows poor adaptation to the target ecology, we recommend using the BC1 strategy. If the aim is to identify transgressive segregants for high HIR, we recommend using the F2 strategy. In either strategy, MAS for *qhir1* may be implemented at an early generation, like on F2 plants or BC1F1 plants with selection of ears from only qhir1+ plants.
(d) At CIMMYT, we follow seed DNA extraction from F2/BC1 seeds for genotyping with *qhir1* markers, which saves substantial time, besides field costs. From the qhir1+ plants, multiple families can be developed while selecting for excellent expression of the *R1-nj* marker and agronomy. In the F3 or BC1F2 generation, a sample of equal number of qhir1+ families (10–15 families) from crosses involving different non-inducer parents can be testcrossed to a tester carrying an easily scorable recessive trait like *lg* to evaluate HIR. Crosses involving the non-inducer parents that showed highest mean HIR in qhir1+ families can be chosen for advancement, while selecting for the *R1-nj* marker expression and agronomic performance.
(e) By the F5 or BC1F4 generations, most agronomically poor families are eliminated and the selected families may be fixed for *R1-nj* marker expression. Hence, selection for HIR can be initiated in these generations by testcrossing the families to recessive testers. In our observation, less than 20% of the qhir1+ families showed HIR similar or higher than the parental inducers. Therefore, we suggest testing at least 50 families involving a non-inducer parent to identify about 10 families with HIR similar or higher than the parental inducer lines.
(f) As HIR in the segregating families may decrease or increase with further inbreeding, we recommend testing the HIR for at least three seasons/environments to assess the stability. Among the 10 lines with higher HIR, one can select a few lines that combine sufficiently high HIR and agronomic performance. Once the final inducer lines are identified, hybrid combinations can be formed using lines from opposite heterotic groups, which can be further evaluated along with the parental inducer lines for assessing their hybrid vigor and HIR, in comparison with the respective parents.

Together, this manuscript presents an efficient approach to develop maize maternal haploid inducers based on MAS and phenotypic selections. 2GTAILs developed through this approach showed high HIR and superior agronomic performance. Use of 2GTAILs improves the efficiency of DH line development for tropical maize breeding programs.

## Author’s Note

Selected 2GTAILs can be availed from CIMMYT by the interested public and private sector organizations. For more information please see https://www.cimmyt.org/second-generation-haploid-inducers-now-available/.

## Author Contributions

VC, SN, and PB designed the experiments. VC, LM, and LL coordinated the field experiments, data collection and sample collection. SN coordinated the genotyping. HU, VC, AM, and SN analyzed the data. VC and SN wrote the manuscript. AM and PB edited the manuscript.

## Conflict of Interest Statement

The authors declare that the research was conducted in the absence of any commercial or financial relationships that could be construed as a potential conflict of interest.

## Footnotes

1 https://plant-breeding.uni-hohenheim.de/84531#jfmulticontent_c167370-2

2 http://www.cimmyt.org/es/que-hacemos/investigacion-sobre-maiz/item/tropicalized-maize-haploid-inducers-for-doubled-haploid-based-breeding

## Abbreviations

DH: Doubled Haploid
EmAR: Embryo Abortion Rate
EnAR: Endosperm Abortion Rate
HIR: Haploid Induction Rate
*lg*: *liguleless*
MAS: Marker-assisted selection
QTL: Quantitative Trait Locus
2GTAILs: Second-generation TAILs
TAILs: Tropically Adapted Inducer Lines
