# Supplementary material for: Marker-Assisted Breeding of Improved Maternal Haploid Inducers in Maize for the Tropical/Subtropical Regions
Source: Front Plant Sci. 2018 Oct 18;9:1527. doi: 10.3389/fpls.2018.01527 (PMC6201356; doi:10.3389/fpls.2018.01527)
Supplement: Supplementary file 1 [file Table_1.PDF]

Supplementary Material

Marker-Assisted Breeding of Improved Maternal Haploid Inducers in Maize for the Tropical/Subtropical Regions

Vijay Chaikam[1], Sudha K Nair[2], Leocadio Martinez[3], Luis Antonio Lopez[3], H. Friedrich Utz[4], Albrecht E Melchinger[4], Prasanna M Boddupalli[1*]

*** Correspondence: Dr. Prasanna M Boddupalli**: b.m.prasanna@cgiar.org

Supplementary Tables

Supplemental Table 1: F1 crosses generated between non-inducers and inducers

Entry	Cross
1	CML451 X (RWSXUH400)
2	CML451 X TAIL7
3	CML451 X TAIL8
4	CML451 X TAIL9
5	CML495 X TAIL7
6	CML495 X TAIL8
7	CML495 X TAIL9
8	CML269 X TAIL8
9	CML269 X TAIL9
10	CML395 X TAIL8
11	CML395 X TAIL9
12	CKL05017 X TAIL9
13	CKL05022 X TAIL9

Supplemental Table 2: Number of plants/families grown and advanced at each generation involving each of the non-inducer parents in (A) the F2 strategy, and (B) the BC1 strategy.

A)

**Non-inducer parents**	**F2**	**F3**	**F4**	**F5***	**F6**	**Candidates**
	**-------------------------------------Selection criterion---------------------------**					
	Agronomy, *R1-nj*	Agronomy, *R1-nj*	qhir1+ and agronomy	HIR	HIR	HIR
CML451	1900	559	202	70	25	0
CML495	1425	393	111	39	15	2
CML269	950	257	83	30	29	6
CML395	950	118	18	11	1	0
CKL05017	475	80	13	6	5	1
CKL 05022	475	51	13	7	0	0
**Total**	**6175**	**1458**	**440**	**163**	**75**	**9**

*included 68 qhir1+ and 95 qhir1- families

B)

**Non-inducer parents**	**BC1F1**	**BC1F2**	**BC1F3**	**BC1F4**	**BC1F5**	**Candidates**
	**---Selection criterion-------------------------------------**					
	Agronomy, *R1-nj*	Agronomy, qhir1+, *R1-nj*	qhir1+ and agronomy	HIR	HIR	HIR
CML451	760	134	109	148	0	0
CML495	570	79	46	37	16	5
CML269	380	56	48	61	137	19
CML395	190	11	11	16	11	2
CKL05017	190	24	20	10	4	1
CKL 0522	190	14	16	0	0	0
**Total**	**2280**	**318**	**250**	**272**	**168**	**27**

Supplemental Table 3: Number of plants/families advanced at each generation involving each of the inducer parents in A) the F2 strategy and B) the BC1 strategy

A)

**Inducer parents**	**F2**	**F3**	**F4**	**F5**	**F6**	**Candidates**
	---Selection criterion---					
	Agronomy, *R1-nj*	Agronomy, *R1-nj*	qhir1+ and agronomy	HIR	HIR	HIR
RWS x UH400	475	124	50	20	4	0
TAIL9	2850	235	177	64	23	2
TAIL8	1900	520	151	59	41	6
TAIL7	950	579	62	20	7	1
**Total**	**6175**	**1458**	**440**	**163**	**75**	**9**

B)

**Inducer parents**	**BC1F1**	**BC1F2**	**BC1F3**	**BC1F4**	**BC1F5**	**Candidates**
	---Selection criterion---					
	Agronomy, *R1-nj*	Agronomy, qhir1+, *R1-nj*	qhir1+ and agronomy	HIR	HIR	HIR
RWS x UH400	190	40	40	79	0	0
TAIL9	1140	139	116	130	117	18
TAIL8	570	78	56	63	51	9
TAIL7	380	61	37	0	0	0
**Total**	**2280**	**318**	**249**	**272**	**168**	**27**

Supplemental Table 4: HIR of candidate inducers assessed in different cycles presented along with mean and standard error (SE).

2GTAIL	Cross	--HIR------------------------------							
		Evaluation Environment							
		MZ14B	MZ15A	MZ16A	AF16A	MZ17A	KI16A	Mean	SE
1	CML495 x TAIL7	5.9	9	3.7	3.3	5.5		5.5	1.01
2	CML495 x TAIL9	6.3	6.3	4.5	2.1	4.6		4.8	0.74
3	CML269 x TAIL8	7.8	6.8	6	6.5	6.3		6.7	0.32
4	CML269 x TAIL8	6	6.3	5.6	4.3	3.4		5.1	0.56
5	CML269 x TAIL8	6.8	4.8	8.1	6.3	5.9		6.4	0.54
6	CML269 x TAIL8	13.6	14.8	11.6	12.1	13	13.2	13.1	0.47
7	CML269 x TAIL8	9.6	10.7	9.8	8.6	10.9	11.3	10.1	0.42
8	CML269 x TAIL8	8.1	12.2	8.9	8.3	8.5	9.3	9.2	0.63
9	CKL05017 x TAIL9	12.5	13.5	9.6	9.9	9.4	10.7	10.9	0.68
101	(CML269 x TAIL9) x CML269	11.9	7.7	6.4	5.8	7	9.7	8.1	0.94
102	(CML269 x TAIL9) x CML269	10.5	9.4	8.6	8.1	8.1	10.5	9.2	0.45
103	(CML269 x TAIL8) x CML269	11.2	10	5.6	1.5	5.7	10.4	7.4	1.54
104	(CML269 x TAIL9) x CML269	10.7	9.7	7.7	6	10.1	10.6	9.1	0.77

105	(CKL05017 x TAIL9) x CKL05017	10.5	8.4	10.5	6.9	7	13.1	9.4	0.99
106	(CML269 x TAIL9) x CML269	9.5	11.8	7.7	5.7	8.9		8.7	1.01
107	(CML269 x TAIL8) x CML269	9.5	10.6	5.4	4.1	6.7	7.4	7.3	1
108	(CML269 x TAIL9) x CML269	9.4	8.8	10.6	7.7	7.6		8.8	0.57
109	(CML269 x TAIL9) x CML269	9.3	11.7	7.1	7.4	8.1	13.2	9.5	1.02
110	(CML269 x TAIL8) x CML269	9.2	6.6	7.8	4.3	8.5	10.8	7.9	0.92
111	(CML269 x TAIL9) x CML269	9.2	8.5	7.1	4.4	9		7.6	0.89
112	(CML269 x TAIL9) x CML269	9.1	8.1	7.3	7.4	6.3		7.6	0.46
113	(CML269 x TAIL9) x CML269	9.1	9	5.8	5.6	5.7	7.3	7.1	0.66
114	(CML269 x TAIL9) x CML269	9	8.9	7.8	6.6	7.4	9.6	8.2	0.46
115	(CML495 x TAIL8) x CML495	8.9	9.3	6.6	4.9	6.3	6.6	7.1	0.66
116	(CML269 x TAIL9) x CML269	8.8	8.4	6.8	4.7	5.1	9.5	7.2	0.82
117	(CML269 x TAIL9) x CML269	8.8	11.2	7.4	4.3	9		8.2	1.15

118	(CML269 x TAIL9) x CML269	8.5	7.3	7.5	5	6.5	7.8	7.1	0.51
119	(CML269 x TAIL8) x CML269	8.4	6.8	7.2	5.3	6.2	8.1	7	0.48
120	(CML269 x TAIL9) x CML269	8.3	5.8	3.9	5.1	5		5.6	0.73
121	(CML395 x TAIL9) x CML395	8.2	8.4	5.8	4.1	6.6	7.1	6.7	0.66
122	(CML395 x TAIL9) x CML 395	7.9	8.1	7.6	8.7	6.7	6.6	7.6	0.32
123	(CML269 x TAIL9) x CML269	8	6.5	6.9	7.5	9	12.7	8.4	0.92
124	(CML495 x TAIL8) x CML 495	7.9	8	5.8	7.9	8.1	10.4	8	0.6
125	(CML495 x TAIL8) x CML 495	7.8	7.9	7	6.7	8.1	7.9	7.6	0.23
126	(CML495 x TAIL8) x CML 495	7.5	7.2	7.9	8.4	6.3		7.5	0.37
127	(CML495 x TAIL8) x CML 495	7.4	8.1	7.9	7	9	8.6	8	0.3

Supplemental Table 5: Assessment of HIR of two best 2GTAILs and their hybrid in populations relevant to maize breeding programs.

**Population**	**----------------------HIR (%)------------------------**		
	2GTAIL006	2GTAIL009	2GTAIL009 x 2GTAIL006
MVA1	15.2	12.1	13.7
MVA2	11.1	9.0	14.2
85C4	10.9	7.4	12.0
1069	14.5	10.1	12.2
MST1	11.8	7.3	9.0
MPP2	16.3	12.5	9.0
MPP1	14.8	9.9	11.1
**Mean†**	**13.5a**	**9.8b**	**11.6c**
**Standard Error**	**0.4**	**0.5**	**0.5**

† Means followed by the same letter are not significantly different at P ≤.05
